# High Strength and Strong Thixotropic Gel Suitable for Oil and Gas Drilling in Fractured Formation

**DOI:** 10.3390/gels11080578

**Published:** 2025-07-26

**Authors:** Yancheng Yan, Tao Tang, Biao Ou, Jianzhong Wu, Yuan Liu, Jingbin Yang

**Affiliations:** 1 Sinopec Southwest Oil & Gas Company Engineering Technology Research Institute, Chengdu 610031, China; yanyancheng_2008@163.com (Y.Y.); tantaochengdu@163.com (T.T.); obiaou@163.com (B.O.); jianzhong_wu168@163.com (J.W.); 2 School of Petroleum Engineering, China University of Petroleum (East China), Qingdao 266580, China

**Keywords:** lost circulation, polymer gel, thermal stability, plugging, self-filling capacity

## Abstract

In petroleum exploration and production, lost circulation not only significantly increases exploration and development costs and operational cycles but may also lead to major incidents such as wellbore instability or even project abandonment. This paper constructs a polymer gel plugging system by optimizing high-molecular-weight polymers, crosslinker systems, and resin hardeners. The optimized system composition was determined as 1% polymer J-1, 0.3% catechol, 0.6% hexamethylenetetramine (HMTA), and 15% urea–formaldehyde resin. Experimental studies demonstrated that during the initial stage (0–3 days) at 120 °C, the optimized gel system maintained a storage modulus (G′) of 17.5 Pa and a loss modulus (G″) of 4.3 Pa. When the aging period was extended to 9 days, G′ and G″ decreased to 16 Pa and 4 Pa, respectively. The insignificant reduction in gel strength indicates excellent thermal stability of the gel system. The gel exhibited superior self-filling capacity during migration, enabling complete filling of fractures of varying sizes. After aging for 1 day at 120 °C, the plugging capacity of the gel system under water flooding and gas flooding conditions was 166 kPa/m and 122 kPa/m, respectively. Furthermore, a complete gel barrier layer formed within a 6 mm wide vertical fracture, demonstrating a pressure-bearing capacity of 105.6 kPa. This system shows good effectiveness for wellbore isolation and fracture plugging. The polymer gel plugging system studied in this paper can simplify lost circulation treatment procedures while enhancing plugging strength, providing theoretical support and technical solutions for addressing lost circulation challenges.

## 1. Introduction

Lost circulation, a particularly hazardous downhole complication during drilling, not only leads to extended operational cycles, the consumption of drilling fluid materials, and incidents such as stuck pipe, kicks, or wellbore instability, but, in extreme cases, can even result in well abandonment. Furthermore, it causes formation damage, subsequently reducing oil and gas productivity, thereby severely constraining the efficiency of hydrocarbon resource development. Current series of lost circulation control (LCC) techniques developed to address fluid loss (such as chemical gel plugging, particulate bridging, etc.) commonly suffer from insufficient process maturity. Effective plugging often requires iterative trial and error to accumulate experience [1]. Millimeter-scale wide fracture channels in naturally fractured formations, due to the lack of self-adaptive plugging material systems, have become a critical bottleneck hindering the advancement of deep oil and gas drilling and completion technologies. Based on the geometric characteristics of the loss channels, fluid loss can be classified into three categories: porous matrix loss (pore throat diameter < 100 μm), fractured formation loss (fracture width 0.1~10 mm), and vugular/cavernous loss (fracture width > 10 mm) [2].

To address different types of loss channels, researchers worldwide have developed various plugging material systems [3,4,5,6]. According to the plugging mechanism, commonly used lost circulation materials (LCMs) can be categorized as follows: bridging LCMs, high fluid-loss LCMs, flexible self-adaptive LCMs, inorganic LCMs, chemical gel LCMs, and swelling LCMs. Compared to traditional plugging systems, polymer gel plugging materials offer several advantages: the gel system is a flowable fluid before gelation with excellent rheological properties; it is not restricted by fracture size, enabling effective plugging of loss channels across different scales. Once entering the loss zone, the gel system exhibits strong retention capability, gelling within fractures and adhering to fracture faces, generating high viscous resistance [7,8]. However, conventional polymer gels are susceptible to dilution by formation water and exhibit significant gravitational settling within wellbores and large fracture spaces, leading to poor vertical retention and difficulty in achieving complete, effective filling, consequently failing to effectively plug the loss zone. Moreover, controlling the injection timing and gelling time of the gel system is challenging, complicating field operations.

Preformed particle gels (PPGs) are gels prepared and processed under surface conditions before being injected into the target formation via injection wells to block high-permeability zones [9]. PPG particles, a type of PPG system, refer to millimeter-sized pre-synthesized gel particles [10]. PPG plugging technology employs ground-pre-polymerized gel particles as the plugging medium. These particles can absorb water and swell underground, migrating to the loss zone under differential pressure, and achieving plugging through the synergistic effects of bridging/stacking and swelling/compaction [11]. In building PPG systems, inorganic rigid particles can be introduced to enhance mechanical properties. Rigid materials form a supporting skeleton, while the gel component fills the interstitial spaces through plastic deformation, forming a dense plugging layer during compaction, thereby significantly improving the overall pressure-bearing capacity of the system [12]. Wang et al. [13] fabricated an Interpenetrating Polymer Network (IPN) gel. This gel utilizes particles with certain stiffness and a network structure as the primary network, interpenetrating and crosslinking with polymer monomers, forming a structure characterized by “internal rigidity and external toughness.” This enhances the structural stability, temperature resistance, and plugging efficiency of the LCM. The elastic modulus increased by 20–70%, temperature resistance improved by 25%, and the plugging efficiency reached 98.9%. Bu et al. [14] developed an LCM with External Flexibility and Internal Rigidity (EFIR material). This synergistic approach leverages the deformability of flexible materials and the skeleton support of rigid materials to construct a high-strength, dense plugging system. The core functional design of EFIR is its core–shell structure, achieved through inverse suspension polymerization of acrylamide (AM), 2-acrylamido-2-methylpropanesulfonic acid (AMPS), and alkyl ketone polymer (NVP) monomers forming the shell, with modified sand as the core. Results show that EFIR maintains significant flexibility and strength during water absorption and swelling, capable of withstanding pressures up to 7 MPa.

In situ crosslinked gel materials involve injecting components such as polymers and crosslinkers into the formation in a fluid state. Crosslinking reactions occur under downhole high-temperature conditions to form a plugging barrier [15]. This material exhibits excellent size compatibility with loss channels; being fluid before gelation, it can enter and plug loss channels of varying sizes under driving forces like differential pressure [16,17,18]. The crosslinking mechanisms between polymers and crosslinkers can be divided into inorganic metal ion crosslinking and organic crosslinking. Taking the chromium acetate/polyacrylamide system as an example, although the gel formed via ionic bonds possesses high initial strength, its low bond energy stability and rapid crosslinking characteristics make it primarily suitable for shallow, low-temperature reservoirs. To control the crosslinking reaction rate, Albonico et al. [19] added malonate/glycolate (retarders) to the polyacrylamide/chromium system. Their study demonstrated that the introduction of retarders weakens the crosslink network density, leading to a decline in the system’s mechanical properties. To meet environmental requirements in oilfield development, researchers are committed to developing low-toxicity alternatives. Moradi-Araghi [20] evaluated the feasibility of phenol substitutes like aspirin and salicylates, but research on formaldehyde alternatives still largely focuses on hexamethylenetetramine (HMTA). HMTA releases formaldehyde through controlled thermal decomposition. The formaldehyde hydrolyzes to form a methylene glycol intermediate, which then reacts with amide groups on the polymer, forming a gel system possessing both high gel strength (storage modulus exceeding 200 Pa) and high-temperature stability (tolerating temperatures over 120 °C). Sengupta et al. [21] developed a PAM/hydroquinone/HMTA composite system. This gel system maintained structural integrity and excellent mechanical properties even after aging for 7 days at 120 °C.

Non-crosslinked gel plugging technology achieves plugging through molecular self-assembly mechanisms, independent of traditional crosslinkers. Representative systems include cement slurries, specialized gels like ZND, and supramolecular LCMs [22,23]. Cement slurry, a widely used inorganic LCM, is often employed to plug severe loss zones like carbonate formations or gravel layers due to its rapid setting characteristics [24]. Specialized gel systems incorporate functional monomers into the polymer backbone through molecular design, enabling the formation of dynamic physical crosslink networks, and achieve synergistic effects by combining with rigid/flexible materials. Leveraging the synergistic mechanisms of structural fluid theory and supramolecular chemistry, Du et al. [25] developed a thermo-responsive temporary plugging agent characterized by sol–gel-sol transition behavior at different temperatures. At low temperatures, the material exists in a sol state, while temperature elevation leads to stable gel formation; however, further heating reverts the gel back to sol. Experimental results indicate the presence of non-covalent interactions between components, which play a significant role in supramolecular gel formation. These findings suggest this system can function as a temperature-induced supramolecular gel material for temporary plugging. Bai et al. [26] synthesized a P(AM-co-AMPS)/SA Double Network (DN) hydrogel via aqueous polymerization, utilizing hydrophobic association crosslinking networks between acrylamide (AM) and lauryl methacrylate (LMA). This hydrogel exhibits excellent temperature resistance and possesses a porous structure. Mechanical testing revealed high tensile strength (110 kPa), fracture elongation (995.31%), good fatigue resistance, and self-recovery performance under multiple cyclic tensile tests. Plugging tests on fracture models with widths of 0.5 and 1 mm demonstrated that the hydrogel can withstand pressures up to 4.5 MPa at 70 °C.

**Figure 1:**
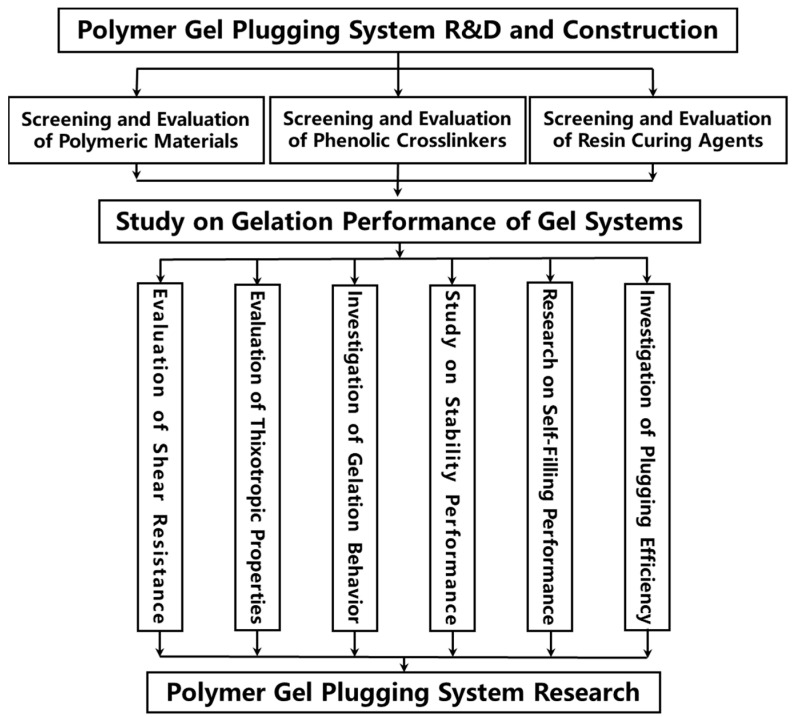
Polymer gel research technical flowchart.

A comprehensive literature survey reveals that gel-based LCM technology demonstrates significant engineering applicability due to its self-adaptive plugging characteristics. However, to enhance the one-trip success rate of lost circulation prevention and control operations under complex formation conditions, it remains necessary to develop novel intelligent gel systems with multi-scale responsiveness (e.g., temperature-sensitive/salinity-sensitive/thixotropic types). Furthermore, innovation in LCM placement techniques is crucial. By synergistically advancing material innovation and process revolution, new theoretical support and technical solutions can be provided to address the challenge of lost circulation. Conventional lost circulation materials (LCMs) often fail to effectively plug due to size mismatch with thief zones or susceptibility to dilution by formation fluids. In wellbores and large-scale fracture spaces, conventional polymer gels are easily diluted by formation water and exhibit significant gravitational settling, resulting in poor longitudinal retention and difficulty in achieving effective, complete filling. To tackle the problem of “ineffective plugging post-gelation due to uneven filling of fracture space” associated with polymer gel plugging materials, this study aims to develop a high-temperature resistant polymer gel plugging system and elucidate its mechanisms for wellbore isolation and fracture plugging, thereby providing theoretical underpinnings and technical countermeasures for solving lost circulation problems. The technical flowchart for polymer gel research is depicted in Figure 1.

## 2. Results and Discussion

### 2.1. Construction of the Polymer Gel System

#### 2.1.1. Optimization of Polymer Type and Concentration

In the construction of gel systems, the structural characteristics of the high-molecular-weight polymer have a decisive influence on system performance. Research indicates that the distribution of functional groups on the polymer backbone directly affects its crosslinking kinetics. Increasing the density of specific functional groups (such as carboxyl and hydroxyl groups) can significantly enhance crosslinking reactivity and the density of the network structure. From the perspective of molecular design, the molecular weight parameters of the polymer are controlled by the monomer conversion rate during polymerization. When monomer conversion increases, the resulting product not only exhibits higher molecular weight but also shows more pronounced chain extension in its molecular conformation [27,28]. The hydrolysis degree of the polymer is positively correlated with its carboxyl group content; a structural characteristic significantly linked to the crosslinking mechanism. The organic phenolic resin crosslinker used in the gel system constructed in this study targets amide groups (−CONH_2_) as active sites. However, due to its hydrolysis degree being within the 10~30% range, its ability to regulate the formation of the gel network is relatively limited. When the polymer hydrolysis degree falls below a critical threshold, it significantly reduces the hydration capacity of the molecular chains, thereby delaying the crosslinking reaction process, manifested as prolonged gelling time for the gel system.

This section primarily investigates four different polymer types: J-1, J-2, J-3, and J-4. Polymer J-1 was synthesized via terpolymerization of acrylamide (AM), 2-acrylamido-2-methylpropanesulfonic acid (AMPS), and N-vinylpyrrolidone (NVP). Its molecular weight distribution ranges from 3.0 × 10^6^ to 5.0 × 10^6^ Da, with a hydrolysis degree controlled between 15~20%. The comparative polymer J-2 is a copolymer of AM and AMPS. Although it maintains a similar molecular weight level (3.0 × 10^6^~5.0 × 10^6^ Da), its hydrolysis degree range is lower (10~15%). Polymers J-3 and J-4 are both low-molecular-weight linear polymers based on acrylamide monomers. Both were produced via controlled hydrolysis processes, resulting in stable hydrolysis degrees of 20~25%, while their molecular weights are significantly lower than those of the first two systems.

**Figure 2:**
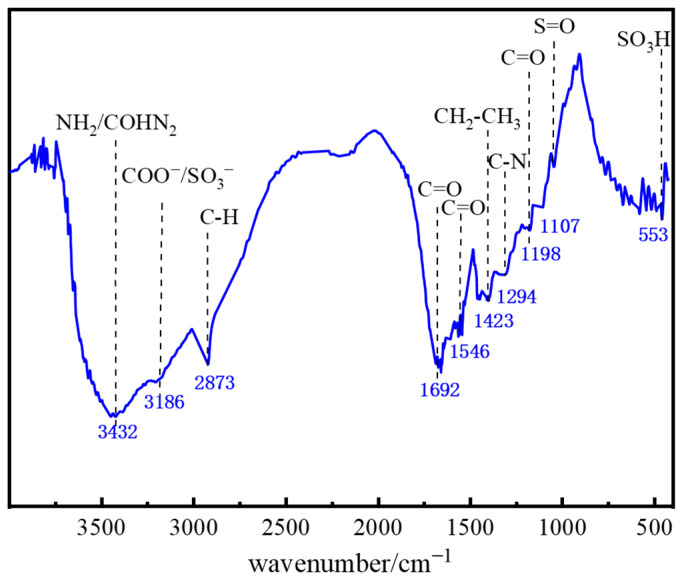
FTIR spectrum of polymer J-1.

As shown in Figure 2, the FTIR spectrum of polymer J-1 exhibits multiple characteristic absorption bands: a broad, strong peak at 3432 cm^−1^ corresponds to the stretching vibration of amino (−NH_2_) or amido (−CONH_2_) groups; the peak near 3186 cm^−1^ is characteristic of carboxylate (−COO^−^) or sulfonate (−SO_3_^−^) groups; the absorption band at 2873 cm^−1^ is attributed to aliphatic C−H bonds. The doublet at 1692 cm^−1^ and 1546 cm^−1^ are typical vibrations of the C=O group in amides; the strong peak at 1423 cm^−1^ is associated with the −CH_2_−CH_3_ group in the AMPS structural unit; the absorption peak at 1294 cm^−1^ corresponds to C−N bond vibration in the NVP structure; the strong peak at 1198 cm^−1^ reveals the presence of the C=O functional group; absorption peaks at 1107 cm^−1^ and 553 cm^−1^ characterize S=O and −SO_3_H group vibrations, respectively. Based on this FTIR analysis, it is confirmed that polymer J-1 possesses structural features of both AMPS and NVP.

**Figure 3:**
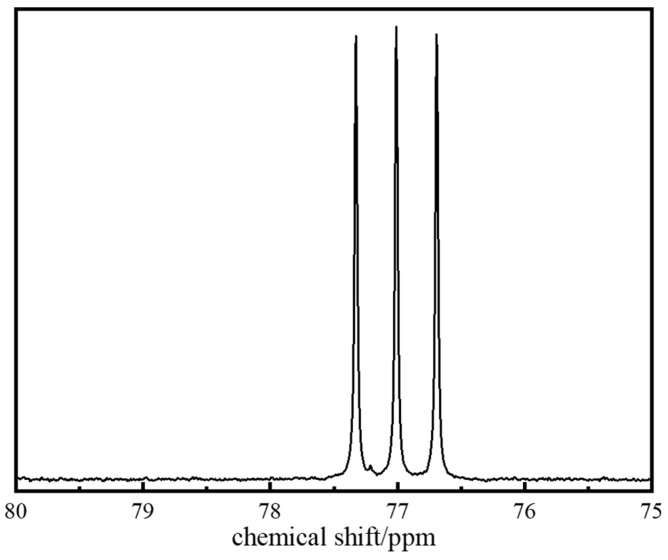
^13^C NMR spectrum of polymer J-1.

**Figure 4:**
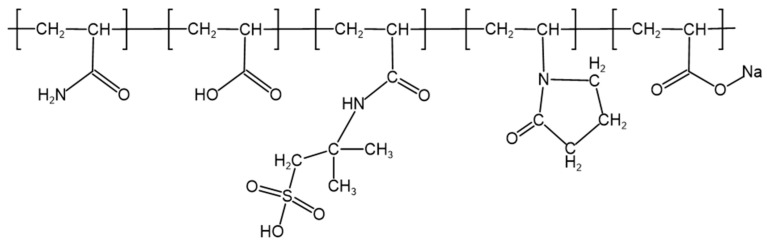
Structural formula of polymer J-1.

To determine the specific chemical composition of polymer J-1, Carbon-13 Nuclear Magnetic Resonance (^13^C NMR) characterization was performed (spectrum shown in Figure 3). Analysis of key characteristic peaks revealed that the methylene group (−CH_2_) in the five-membered ring coordinated to the carbonyl group of the NVP unit produces a characteristic resonance peak at δ = 77.8 ppm, while the methyl group (−CH_3_) in the AMPS structure corresponds to a significant signal at δ = 76.1 ppm. By cross-validating the chemical shift characteristics of each functional group, the molecular structure model of copolymer J-1 was successfully established (Figure 4).

**Figure 5:**
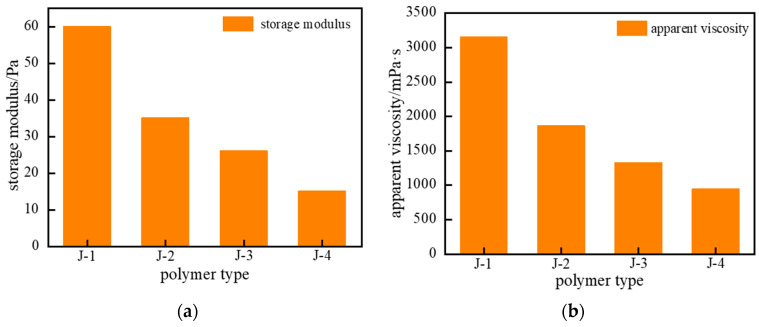
Schematic comparison of temperature resistance for different polymer types: (**a**) Storage Modulus (G′); (**b**) Apparent Viscosity.

Based on the above analysis, the gelation performance of the four polymer types (J-1, J-2, J-3, and J-4) was examined. The concentration of hexamethylenetetramine (HMTA) was fixed at 0.6%, catechol at 0.3%, and urea–formaldehyde resin at 15%. The polymer gel systems were synthesized at 120 °C. As shown in Figure 5, polymer J-2 solution itself exhibits poor temperature resistance, making it difficult to synthesize a high-strength polymer gel. When polymer J-3 was used as the primary polymer in the gel system and aged for 3 h under high temperature, the system viscosity increased moderately, but the overall gel strength remained weak. Using polymer J-4 as the primary gel polymer resulted in a partial improvement in gel strength, but it was still insufficient. Under high-temperature conditions, conventional partially hydrolyzed polyacrylamide (HPAM)-based gel systems are prone to structural failure, primarily attributed to the synergistic effects of thermal degradation and hydrolysis of the polymer backbone. Consequently, they cannot effectively crosslink with crosslinkers to form a gel system of adequate strength. In contrast, the gel formed using polymer J-1 as the primary component exhibited higher gel strength, higher apparent viscosity, and maintained good structural integrity. Therefore, J-1 was selected as the primary polymer for the thixotropic polymer gel isolation system.

**Figure 6:**
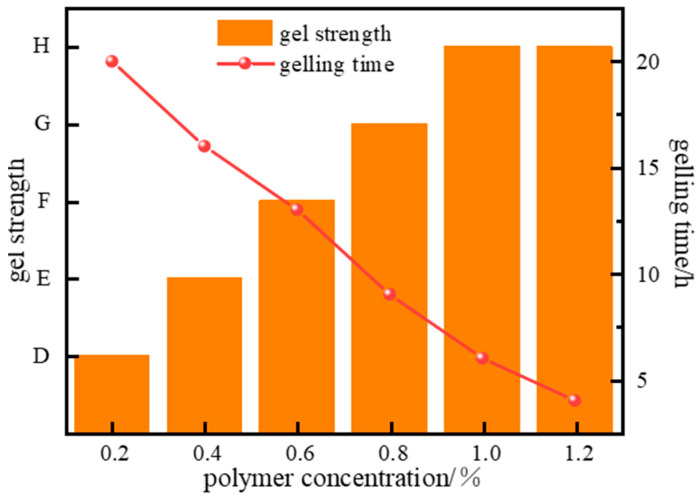
Effect of J-1 concentration on gel strength.

The concentration of the high-molecular-weight polymer has a decisive impact on gel system performance. Excessively low polymer concentration results in insufficient spatial distribution density of molecular chains to support the formation of effective crosslinking points, leading to a discontinuous network structure, difficulty in gelation, or very low gel strength post-gelation. Excessively high polymer concentration causes a sharp increase in apparent viscosity, leading to non-linear growth in shear stress. This directly affects the flow characteristics of the gel solution during injection and can cause rapid gelation, making it difficult to pump the system in a fluid state to the target downhole location. To address these challenges, the effect of polymer concentration variation (0.2~1.2%) on the gelation performance of the system was further investigated. Gelation experiments (Figure 6) show that the gelling time of the system gradually shortens as polymer concentration increases. As the polymer concentration rises from 0.2% to 1.0%, the gelling time decreases from 20 h to 4 h.

As the polymer concentration increases, the number of available crosslinking sites rises, accelerating the crosslinking reaction rate and enhancing the strength of the gel system. However, since the number of crosslinking sites provided by the crosslinker at a fixed concentration is limited, increasing the polymer concentration beyond a certain point yields no significant further improvement in system strength or stability. Experimental results indicate that gel strength increases with rising polymer concentration. However, increasing the concentration further to 1.2% did not increase gel strength, indicating that the polymer at 1.0% concentration completely reacts with the crosslinker.

**Figure 7:**
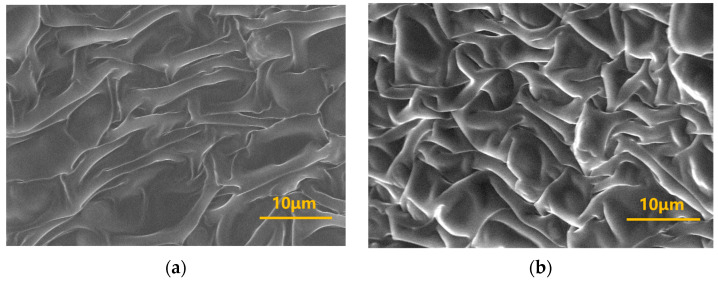
Microstructure of gel systems at different J-1 concentrations: (**a**) 0.4% J-1; (**b**) 1.0% J-1.

Environmental Scanning Electron Microscopy (ESEM) was employed to characterize the three-dimensional (3D) network microstructure of gels formed by crosslinking different concentrations of polymer J-1 (0.4%, 1.0%) with the organic phenolic resin crosslinker system (HMTA-catechol). This provides a micro-mechanistic analysis of the gelation behavior. The microstructure images (Figure 7) reveal that the density of the 3D network in the gel system significantly increases with higher J-1 concentration, and the size of most meshes within the network structure also decreases. This phenomenon occurs because an increase in polymer concentration leads to a corresponding increase in the number of amide groups (−CONH_2_) on the polymer chains. This increases the total number of active crosslinking sites between the polymer molecules and the phenolic crosslinker, accelerating the crosslinking reaction rate. Consequently, the resulting gel exhibits a denser 3D mesh structure and significantly higher gel strength.

#### 2.1.2. Optimization of Crosslinker Type and Concentration

In the construction of gel systems, the crosslinker, as the core functional component for forming the three-dimensional gel network, significantly influences the gelation performance. While organic phenolic crosslinkers are widely used in polymer gel systems, traditional phenol-based systems present ecological toxicity concerns [29,30]. Guided by green chemistry principles, this study compares gelling time, gel strength, and long-term stability among polymer gels prepared with different crosslinkers, analyzing the impact of various phenol and aldehyde crosslinkers on the gelation performance of high-molecular-weight polymer gel systems.

##### Optimization of Aldehyde Crosslinker

This section analyzes the effect of different aldehyde crosslinker types on the gelation performance. The concentration of polymer J-1 was fixed at 1.0%, the phenolic crosslinker catechol at 0.3%, and urea–formaldehyde resin at 15%. The concentration range of different aldehyde crosslinkers was 0.1~0.6%. Gel system solutions were prepared using deionized water. The influence of aldehyde crosslinker type and concentration on gelling time and gel quality of the polymer gel system was evaluated at 120 °C. Experimental results are presented in Table 1.

The gelation performance of the thixotropic polymer gel system was further investigated at 120 °C to assess the long-term stability impact of different aldehydes. Results in Table 1 show that when formaldehyde was used as the aldehyde crosslinker, gelling time was very short, reaching full gelation within approximately 1 h. This is primarily because formaldehyde rapidly participates in the crosslinking reaction, leading to a sharp increase in gel strength grade. However, the excessively fast reaction rate hinders the formation of a dense crosslinked structure between the polymer and crosslinker. Moreover, after aging for only 6 h at high temperature, severe polymer dehydration and degradation occurred. Paraformaldehyde exhibited significant structural limitations as a crosslinker in the gelation process. Although it can release active formaldehyde through hydrolysis at high temperature, its release rate is mismatched with the crosslinking reaction rate. Furthermore, the polymer backbone undergoes chain scission and functional group degradation under thermal stress, severely compromising the activity of effective crosslinking sites. The sustained-release characteristics of paraformaldehyde at 120 °C prevent the establishment of a stable crosslinked framework before polymer chain breakdown and hydrolysis, ultimately resulting in no gel formation. Complete hydrolysis was observed after 24 h of aging. Therefore, formaldehyde and paraformaldehyde systems are unsuitable for constructing thixotropic polymer gel isolation systems.

Finally, the gelation performance using HMTA as the aldehyde crosslinker was evaluated. With polymer J-1 fixed at 1.0%, catechol at 0.3%, and urea–formaldehyde resin at 15%, the effect of HMTA concentration (0.2~0.8%) on gelation performance was investigated at 120 °C. Results showed that polymer gels with significant strength formed across the entire HMTA range tested, with gelling times between 6 and 15 h.

Further evaluation of HMTA’s impact on thermal stability revealed that at an HMTA dosage of 0.6%, the system exhibited less than 10% dehydration after aging for 7 days at 120 °C, demonstrating excellent thermal stability. The system also maintained stable viscoelastic characteristics under sustained high temperature, with the storage modulus loss rate reduced by 62% compared to traditional crosslinking systems, indicating superior stability of the crosslinked network.

**Figure 8:**
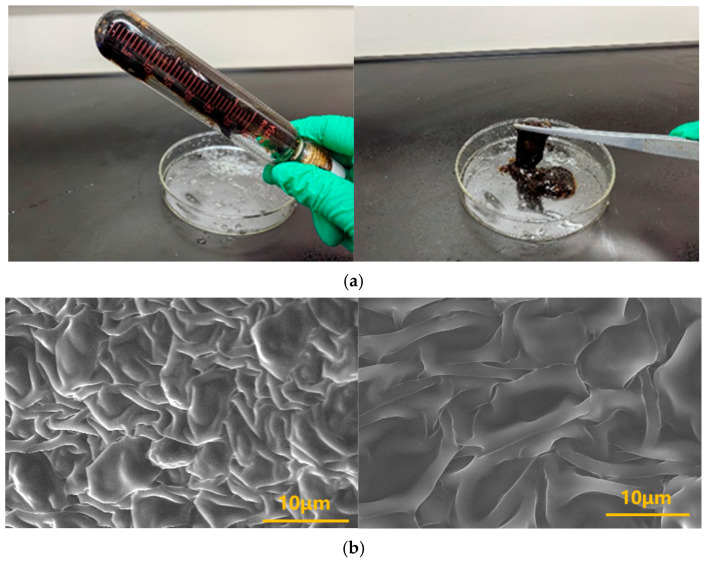
Schematic diagram of gel formation for the polymer J-1/hexamethylenetetramine-catechol polymer gel system: (**a**) Physical state of gel; (**b**) Microscopic network structure of gel.

To elucidate the thermal stabilization mechanism, Environmental Scanning Electron Microscopy (ESEM) was employed to characterize the microstructure of the gelled network. Results (Figure 8) show that the gel sample maintained a continuous, interpenetrating 3D network structure even after prolonged high-temperature aging. This structural characteristic enables the gel system to inhibit the expulsion of most water molecules through a mesh-locking effect under downhole high-temperature and high-pressure conditions, contributing to its exceptional thermal stability.

**Figure 9:**
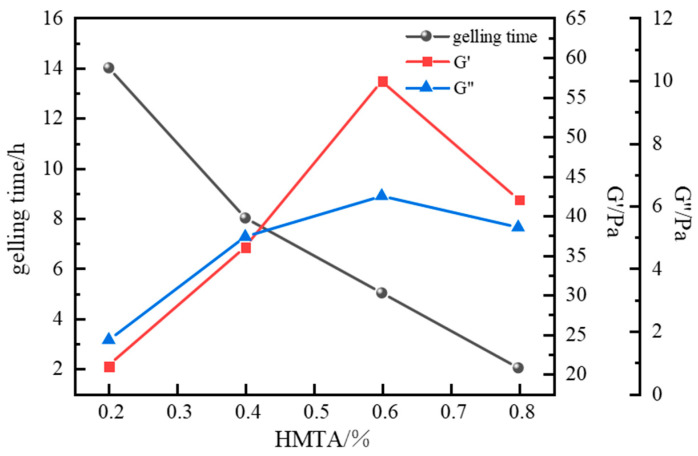
Effect of HMTA concentration on gelling time and storage modulus.

In high-temperature crosslinking kinetic studies, HMTA’s thermal decomposition kinetics exhibited a moderate formaldehyde release rate. This controlled release mechanism effectively balances the crosslinking reaction process with polymer chain thermal stability. Sydansk bottle testing confirmed that gels formed by this system possess excellent integrity. Consequently, HMTA was selected as the optimal aldehyde crosslinker. With polymer J-1 fixed at 1.0%, catechol at 0.3%, and resin hardener at 15%, the influence of HMTA concentration (0.2~0.8%) on system performance was further investigated. Results are shown in Figure 9.

Results indicate that HMTA concentrations ranging from 0.2% to 0.8% successfully crosslinked with the polymer at 120 °C, forming intact gel systems of varying strengths. HMTA concentration exhibited a significant negative correlation with gelling time, with overall gelling times ranging from 2 to 14 h. At an HMTA concentration of 0.2%, crosslinking was insufficient, forming only a weak primary network with a storage modulus (G′) of 21 Pa and a loss modulus (G″) of 1.7 Pa. As HMTA concentration increased to 0.6%, the density of active crosslinking sites substantially increased, significantly enhancing gel strength. The G′ and G″ reached 57 Pa and 6.3 Pa, respectively, indicating excellent viscoelastic properties. However, a performance inflection point occurred beyond 0.6% concentration, resulting in decreased gel strength. This decline is attributed to excessive crosslinking at high crosslinker concentrations. The drastically increased reaction rate leads to over-crosslinking, increasing the proportion of network defects and hindering the formation of a stable crosslinked structure. Based on the principle of performance optimization, an HMTA concentration of 0.6% was selected.

##### Optimization of Phenolic Crosslinker

This section analyzes the influence of phenolic crosslinkers (phenol, hydroquinone, catechol) on polymer gelation performance. Using terpolymer J-1 (concentration range 0.2~1.0%) as the primary gel agent, HMTA at 0.6%, and urea–formaldehyde resin at 15%, the effect of phenolic crosslinker type and concentration on gel system performance was evaluated at high temperature. Results are presented in Table 2.

As shown in Table 2, using phenol as the crosslinker resulted in incomplete gels with insufficient strength for plugging requirements. Systems using hydroquinone formed distinct gels with gelling times between 8 and 15 h, achieving strength Grade D. However, their long-term stability was poor. Increasing the J-1 polymer concentration to 0.8% reduced the gelling time to 10 h, and a further increase to 1.0% decreased it to 7 h. Mechanistic analysis suggests that increased molecular chain entanglement density promotes crosslinking reaction kinetics. Although gelling time decreased, gel strength significantly increased due to enhanced efficiency in constructing the 3D network.

**Figure 10:**
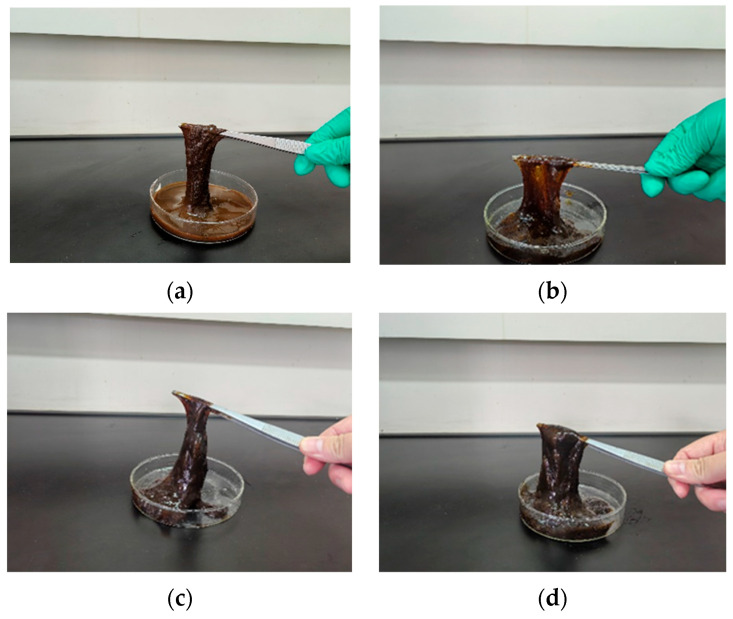
Gel state at different catechol concentrations: (**a**) 0.1% catechol; (**b**) 0.2% catechol; (**c**) 0.3% catechol; (**d**) 0.4% catechol.

**Figure 11:**
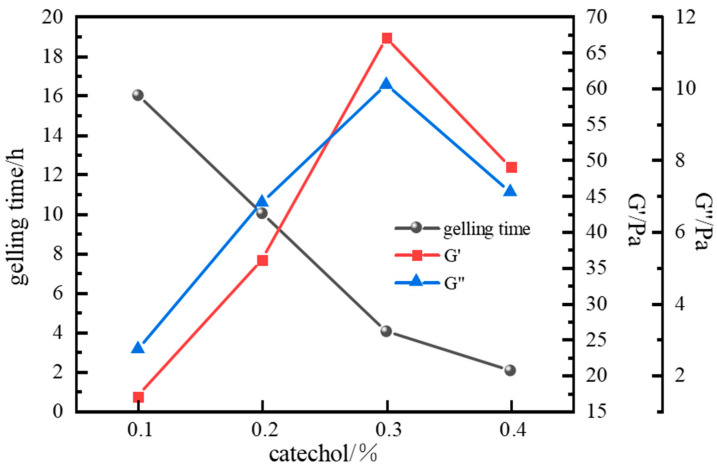
Effect of catechol concentration on gelling time and storage modulus.

When catechol was used as the phenolic crosslinker, the resulting gels exhibited higher strength than those formed with phenol or hydroquinone. The gel system demonstrated the best wall-adhering ability and maintained excellent long-term stability, showing less than 10% dehydration after aging for 7 days at a high temperature. Therefore, catechol was selected as the optimal phenolic crosslinker. The influence of catechol concentration on gelation was further investigated. Results (Figure 10 and Figure 11) show that gelling time decreased with increasing catechol concentration, while gel strength initially increased and then decreased. At a catechol concentration of 0.3%, gel strength reached its maximum, with G′ and G″ values of 67 Pa and 10.1 Pa, respectively. The gel also exhibited high viscosity and excellent wall-adhering performance. However, when the phenolic crosslinker concentration exceeded 0.3%, the complex modulus decreased, and gel strength weakened. This observation aligns with the phenomenon seen in the aldehyde crosslinker optimization study; excessive crosslinker concentration leads to over-crosslinking and performance degradation. Consequently, an optimal catechol concentration of 0.3% was selected.

#### 2.1.3. Optimization of Resin Hardener Type and Concentration

To enhance the long-term stability of the thixotropic polymer gel system under high-temperature downhole conditions, urea–formaldehyde resin was introduced as a hardener. The self-polymerization curing reaction of urea–formaldehyde resin at elevated temperatures is pivotal for improving the thermal stability of the gel plugging agent. Functional groups in urea–formaldehyde molecules—including −CH_2_OH (hydroxymethyl), −NH− (imino), and −NH_2_ (amino)—exhibit high reactivity [31]. These groups undergo polycondensation reactions: hydroxymethyl groups form ether bonds (−CH_2_−O−CH_2_−) via dehydration condensation; amino and hydroxymethyl groups react to generate methylene bridges (−CH_2_−NH−).

These reactions trigger continuous interweaving between urea–formaldehyde molecules, gradually constructing a three-dimensional (3D) network structure. This network significantly enhances the gel strength and viscoelasticity of the system. Consequently, it improves the gel’s temperature resistance and long-term stability, thereby boosting the pressure-bearing capacity of the gelled plugging agent for plugging large fracture loss channels post-gelation under high temperatures.

In high-temperature downhole environments, gel plugging systems may experience polymer chain scission and alterations in molecular aggregation states due to thermal effects, compromising gelation performance. The 3D skeletal network formed by urea–formaldehyde resin effectively restricts disordered molecular motion within the gel system. Simultaneously, this 3D structure reinforces the system’s pressure-bearing capacity, enabling it to withstand formation pressures under high-temperature conditions. However, insufficient urea–formaldehyde resin addition may result in an incomplete 3D network structure. Reduced crosslinking points between molecules lead to inadequate gel system strength. Under these conditions, the gel plug formed by the gel agent is prone to deformation or even rupture, potentially failing to withstand excessive deep formation pressures or formation fluid scouring forces, ultimately impairing plugging effectiveness.

Increasing the urea–formaldehyde resin dosage increases the number of reactive functional groups, yielding a denser 3D network structure. The strength of the gel plug gradually increases, enabling better resistance to external pressure and fluid scouring, and more effective plugging of formation pores and fractures. However, excessive urea–formaldehyde resin can cause the gel to become overly hard and brittle. This over-crosslinking reduces gel elasticity, making it susceptible to cracking under external impact, which also diminishes plugging effectiveness. Furthermore, increasing the resin dosage accelerates the curing reaction rate. More reactive functional groups interact within a shorter timeframe, forming crosslinked structures; however, the curing reaction may become excessively rapid and difficult to control.

**Figure 12:**
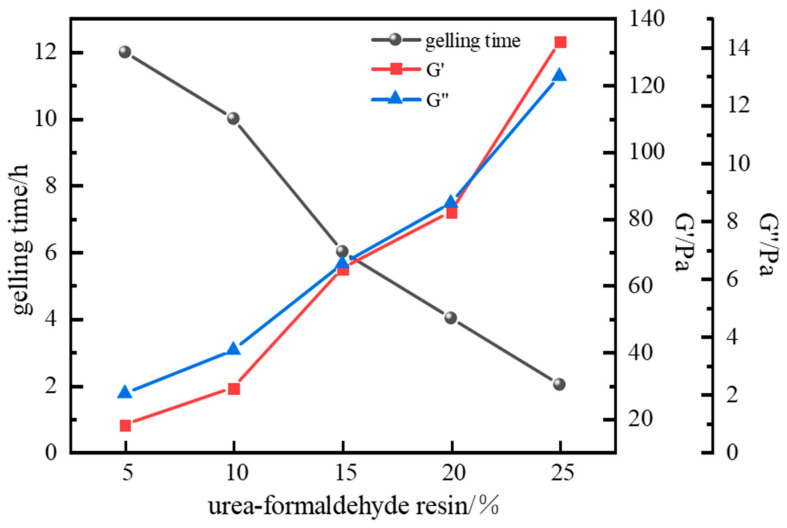
Effect of urea–formaldehyde concentration on gelling time and storage modulus.

The influence of urea–formaldehyde resin hardener dosage on gel strength was investigated using fixed parameters: 1.0% polymer J-1, 0.6% crosslinker hexamethylenetetramine (HMTA), 0.3% catechol, and a gelation temperature of 120 °C. Experimental results (Figure 12) show that urea–formaldehyde resin concentration significantly impacts gel system strength and gelling time. Gelling time decreased from 12 h to 2 h as resin concentration increased, while gel strength continuously increased. At a resin concentration of 15%, the storage modulus (G′) and loss modulus (G″) reached 65 Pa and 6.4 Pa, respectively, meeting isolation strength requirements. Excessively high resin concentrations (>15%) degraded the gel’s viscoelasticity, causing significant gel dehydration, reduced gel toughness, and excessively short gelling times, making the curing process difficult to control. Therefore, a urea–formaldehyde resin concentration of 15% was selected as optimal.

#### 2.1.4. Molecular Structure Characterization of the Polymer Gel System

**Figure 13:**
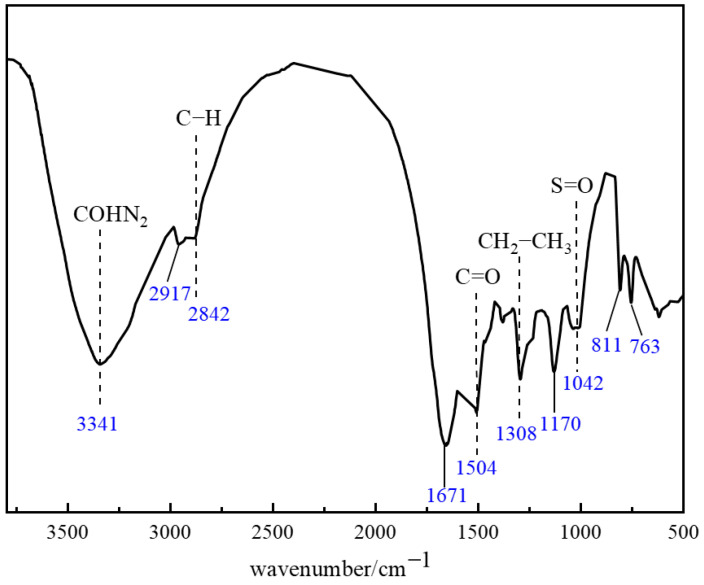
FTIR structural characterization of the polymer gel system.

Fourier Transform Infrared Spectroscopy (FTIR) was employed to characterize the molecular structure of the thixotropic polymer gel system. Based on the above experiments, the optimized formula for the polymer gel system is as follows: 1% polymer J-1 + 0.3% catechol + 0.6% hexamethylenetetramine (HMTA) + 15% resin hardener. Vibration information in the fingerprint region (1350–400 cm^−1^) and functional group region (4000–1350 cm^−1^) revealed characteristic chemical bonding. As shown in Figure 13, the broad, intense absorption peak at 3341 cm^−1^ corresponds to the stretching vibration of amino (−NH_2_) or amido (−CONH_2_) groups. The characteristic peak at 2917 cm^−1^ is attributed to the asymmetric vibration of carboxylate (−COO^−^) or sulfonate (−SO_3_^−^) groups. The typical vibration signal of aliphatic C−H bonds appears at 2842 cm^−1^, while the dual peaks at 1671 cm^−1^ and 1504 cm^−1^ collectively confirm the C=O stretching vibration in amide groups. The strong peak at 1308 cm^−1^ corresponds to the deformation vibration of methylene/methyl (−CH_2_−CH_3_) groups. Significant absorption at 1170 cm^−1^ indicates the presence of C=O functional groups, and the peak at 1042 cm^−1^ relates to S=O bond vibrations. In the low-frequency region, the characteristic doublet splitting at 811 cm^−1^ and 763 cm^−1^ represents the distinctive in-plane S−O−H bending vibration of sulfonic acid groups (−SO_3_H).

FTIR characterization confirmed that synergistic hydrogen bonding and covalent crosslinking between the phenolic crosslinker and polymer J-1 established a multi-scale interactive 3D network. This structure endows the gel system with unique thixotropic responsiveness and thermal stability. Analysis of characteristic peak shifts–specifically, altered vibration modes of phenolic hydroxyl groups (3200–3600 cm^−1^) and amide groups (1671 cm^−1^)–demonstrated intermolecular hydrogen bonding and covalent crosslinking reactions. These results verify the successful synthesis of the target thixotropic polymer gel system, offering molecular-level mechanistic insights for subsequent rheological studies and engineering applications.

Nuclear Magnetic Resonance (NMR) analysis is critical for characterizing the crosslinked network of thixotropic polymer gels. NMR leverages the spin properties of atomic nuclei, where nuclei in different chemical environments absorb radiofrequency radiation at distinct frequencies under a magnetic field. Excited nuclear spin systems undergo energy level transitions, generating detectable induced electromotive force signals. Parameters, including chemical shift, peak splitting, and integration area, provide detailed molecular structural information about the gel system. For crosslinked polymer gels, NMR reveals atomic environments near crosslinking sites, enabling assessment of crosslinking density and structure through signals from specific atoms in crosslinkers.

**Figure 14:**
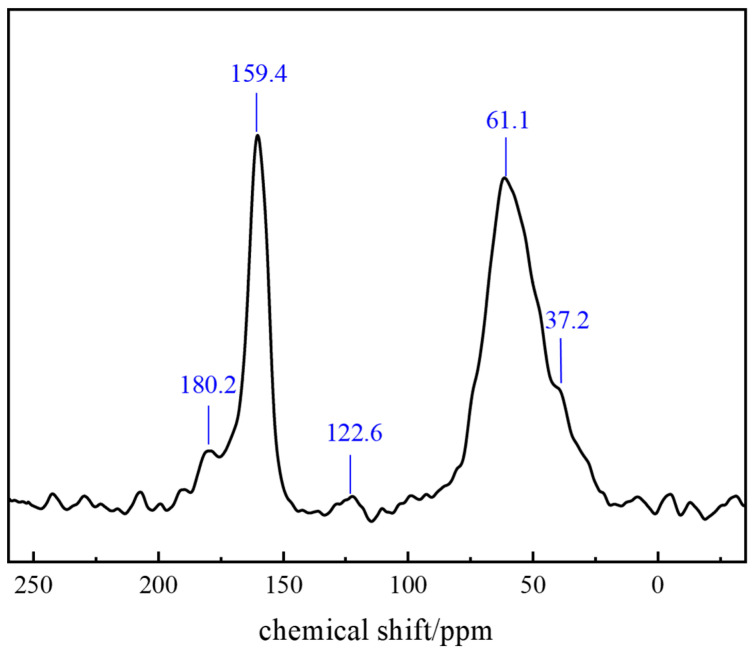
NMR analysis of the polymer gel system.

Carbon-13 Nuclear Magnetic Resonance (^13^C-NMR) is based on the magnetic resonance phenomenon of ^13^C nuclei. Under a magnetic field, ^13^C nuclei generate distinct energy levels. Upon excitation by radiofrequency pulses, transitions between these levels produce NMR signals. The frequency of these signals correlates with the chemical environment of the ^13^C nuclei, measured and converted into chemical shift (δ in ppm). As a core parameter, chemical shift reflects the carbon atom electron density distribution, providing structural information about different carbon atoms in the polymer gel system. ^13^C-NMR analysis of the gel (Figure 14) determined its specific chemical structure as follows: the peak at δ = 180.2 ppm corresponds to the C=O bond in amide groups; δ = 159.4 ppm to aromatic carbon atoms in benzene rings; δ = 61.1 ppm to −NH_2_ or −CONH_2_ groups in the polymer structure; and δ = 37.2 ppm to the methyl group (−CH_3_) in AMPS (2-acrylamido-2-methylpropanesulfonic acid) units.

### 2.2. Study on Gelation Performance of Polymer Gel Systems

#### 2.2.1. Shear Resistance of Polymer Gel System

The apparent viscosity (η) of the material decreases with increasing temperature (T) within a defined range, while at any given temperature, it declines with rising shear rate (γ).

This rheological behavior can be mathematically described by the power-law model for pseudoplastic fluids:

H = Kγ^η−1^, (1)

where K represents the consistency coefficient (Pa·sⁿ), n denotes the flow behavior index (dimensionless), and γ is the shear rate (s^−1^).

**Figure 15:**
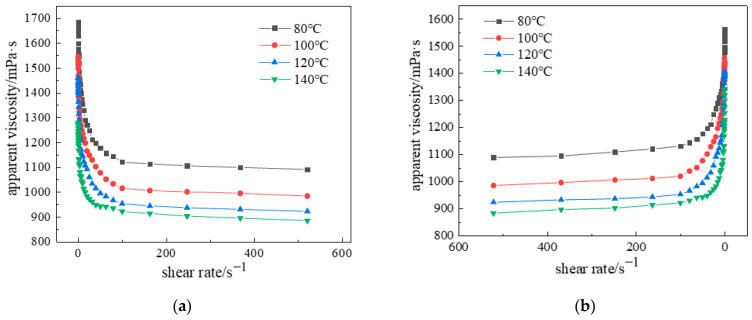
Apparent viscosity versus shear rate at different temperatures: (**a**) Forward shear; (**b**) Reverse shear.

Both K and n exhibit significant temperature dependence, with K typically decreasing at elevated temperatures, demonstrating the complex interdependence between apparent viscosity, temperature, and shear rate. As shown in Figure 15, the influence of shear rate on the gel system’s rheological properties was systematically investigated across 80–120 °C, gel viscosity progressively decreased with increasing shear rate until stabilizing beyond approximately 20 s^−1^. This phenomenon occurs because entangled polymer chains enhance viscosity under static conditions, while applied shear forces disentangle chains and align molecular structures, reducing intermolecular resistance and lowering viscosity. Beyond critical shear rates, disruption of disordered molecular clusters minimizes further viscosity changes.

Experimental measurements revealed an apparent viscosity of 1693 mPa·s at 80 °C and 0.1 s^−1^, decreasing to 1471 mPa·s under identical shear conditions at 120 °C. At 100 s^−1^, viscosity measured 1137 mPa·s (80 °C) and 929 mPa·s (120 °C), with minimal reduction indicating exceptional shear resistance. Figure 15b demonstrates the system’s thixotropic recovery, where viscosity increased sharply when shear rate decreased below 50 s^−1^, nearly returning to initial values. This enables practical field application through injection at high shear rates and retention in target zones under low shear conditions. The inverse correlation between temperature and viscosity arises from intensified molecular motion and weakened intermolecular forces. Critically, at 120 °C and 100 s^−1^, apparent viscosity remained above 950 mPa·s, representing 81.37% retention versus the 80 °C baseline and confirming superior thermal stability under extreme downhole conditions.

#### 2.2.2. Thixotropic Behavior of Polymer Gel System

In rheological characterization, thixotropy reflects the dynamic equilibrium response of a material’s network structure under shear fields. Specifically, when shear stress is applied, the deconstruction rate of the three-dimensional network exceeds its reorganization rate, resulting in a hysteresis phenomenon in the viscoelastic response. During gelation, the system reconstructs its network through crosslinking interactions between polymer segments and crosslinkers. This microstructural evolution can be quantitatively analyzed via thixotropic loop testing under dynamic oscillatory shear.

To determine whether a polymer solution exhibits thixotropy, the following cyclic shear experiments are conducted: the shear rate is gradually increased and then decreased to obtain a shear stress versus shear rate curve. During cyclic shearing, if the ramp-up curve (shear rate increasing from 0) and ramp-down curve (shear rate decreasing from maximum to 0) do not coincide but form a closed loop, this loop is termed a hysteresis loop. Thixotropic fluids exhibit this hysteresis due to asymmetric structural breakdown and rebuilding. During ramp-up, the increasing shear rate progressively disrupts the internal structure, raising shear stress; however, continuous structural breakdown reduces resistance, diminishing the rate of stress increase. During ramp-down, structural rebuilding begins, reducing shear stress, but the time-dependent reorganization causes the ramp-down curve to fall below the ramp-up curve, forming the hysteresis loop.

**Figure 16:**
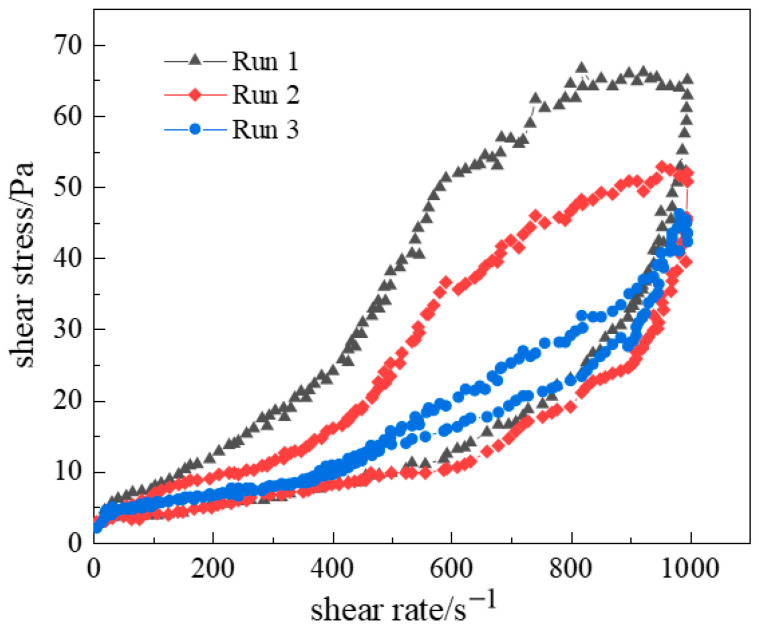
Hysteresis regions during cyclic shear testing.

Different shear rate ranges yield distinct hysteresis loop areas. Testing at lower shear rates causes minor structural disruption, producing smaller loops. Higher shear rates induce severe structural damage, enlarging the loop area. Shear history is also critical: pre-sheared fluids may exhibit altered internal structures. Cyclic shear tests on the optimized gel solution (Figure 16) revealed that the first cycle’s hysteresis loop was significantly larger than subsequent cycles. After three cycles, hysteresis areas progressively diminished, and ramp-up/down curves nearly overlapped. This occurs because long-chain polymer molecules initially entangle in solution. During shearing, entanglements disentangle as the shear rate rises. Irreversible chain stretching or bond breakage during cyclic shear prevents full re-entanglement, reducing the area of thixotropy and hysteresis.

#### 2.2.3. High-Temperature Gelation Performance

**Figure 17:**
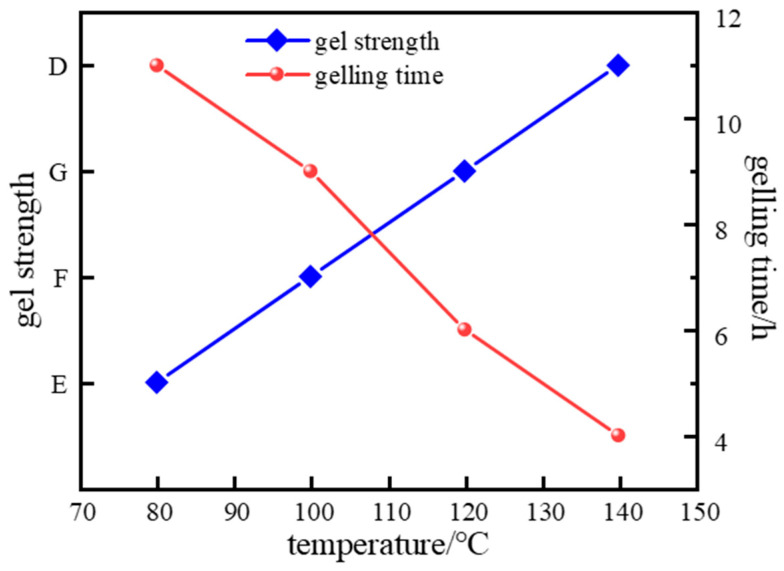
Effect of different temperatures on the gelatinizing properties of the gel system.

Gelling time of polymer gel systems is significantly influenced by temperature. Elevated temperatures generally accelerate chemical reactions. For chemically crosslinked gels (e.g., those formed by polymer–crosslinker polycondensation), high temperature increases reaction rates between polymer molecules and crosslinkers by enhancing collision frequency, expediting crosslinking. Temperature also critically affects gel strength. Higher temperatures promote polymer chain extension and stronger intermolecular interactions. At optimal temperatures, enhanced chain entanglement and crosslinking improve gel strength. However, excessive temperatures may degrade the 3D network; intense molecular motion can break chemical bonds and crosslinking points. To evaluate temperature effects (80–140 °C) on gelling time and strength, experiments used the optimized formulation as a high-temperature in situ crosslinked polymer gel system (Figure 17).

**Figure 18:**
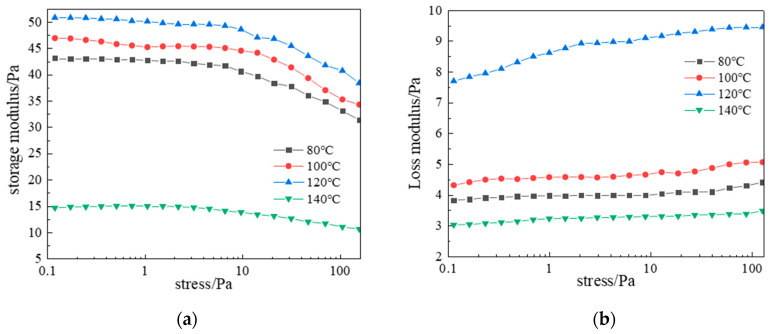
Effect of temperature on complex modulus: (**a**) Storage modulus; (**b**) Loss modulus.

Data in Figure 17 show gelling time decreased from 11 h (80 °C) to 4 h (140 °C). Figure 18 reveals that storage modulus (G′) varied considerably with temperature and decreased with stress, while loss modulus (G″) increased with stress but showed smaller temperature variations. Under identical stress, G′ consistently exceeded G″, confirming superior elasticity essential for effective loss-zone plugging. At 120 °C, optimal crosslinking achieved maximum complex modulus, with G′ and G″ stabilizing at 50 Pa and 9.5 Pa, respectively, indicating a high-strength 3D gel network with optimal viscoelasticity. At 140 °C, accelerated molecular motion reduced crosslinking density and gel strength.

#### 2.2.4. High-Temperature Stability of Polymer Gel System

**Figure 19:**
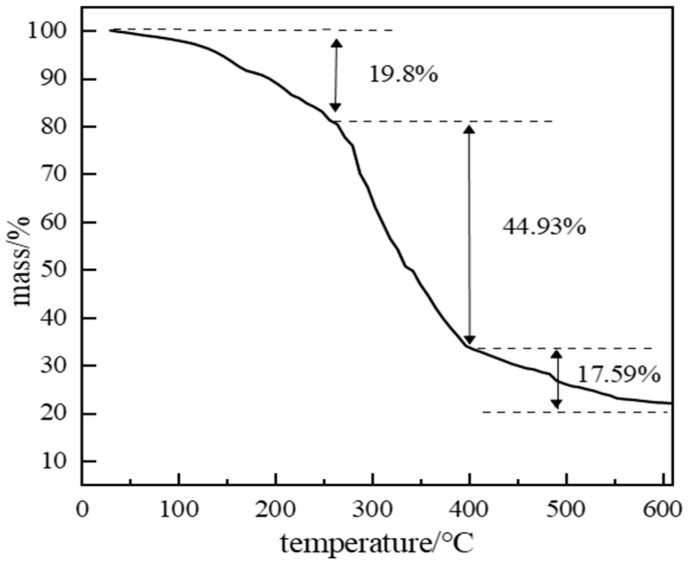
TGA curve of polymer gel material.

Polymer gel isolation systems require long-term stability at formation temperatures to effectively plug high-permeability channels. In high-temperature environments, gels may undergo syneresis (water expulsion) and network degradation. If decomposition occurs, plugging capability is lost. Thermogravimetric analysis (TGA) assessed thermal stability (Figure 19). Between 0 and 150 °C, a 19.8% mass loss occurred due to volatile components consisting primarily of water solvent from the system, along with trace amounts of catechol vapor, formaldehyde, and ammonia generated from the initial decomposition of hexamethylenetetramine. A significant inflection point at 250 °C marked the onset of rapid mass loss, indicating backbone scission and network disintegration. This defines the thermal stability limit; below 250 °C, the gel maintains structural integrity.

**Figure 20:**
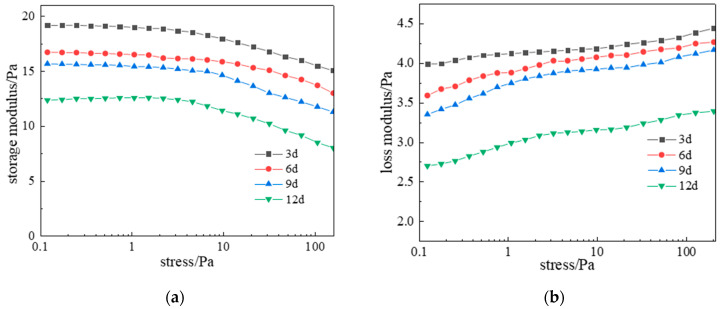
Effect of aging time on complex modulus at 120 °C: (**a**) Storage modulus; (**b**) Loss modulus.

For deep high-temperature reservoir applications, long-term network stability is critical. Gel samples aged at 120 °C for 3, 6, 9, and 12 days underwent dynamic rheometry and microstructural characterization. Results (Figure 20) show viscoelastic properties gradually weakened with aging time. Initially (0–3 days), G′ and G″ stabilized at 17.5 Pa and 4.3 Pa. After 9 days, they decreased marginally to 16 Pa and 4 Pa, demonstrating exceptional long-term stability under high-temperature conditions.

#### 2.2.5. Self-Filling Capacity of Polymer Gel Systems

**Figure 21:**
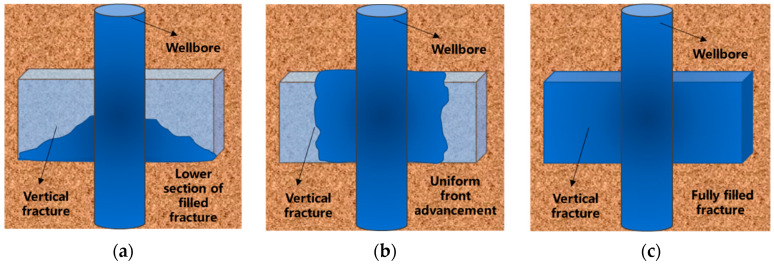
Self-adaptive uniform fracture filling process: (**a**) Bottom filling; (**b**) Uniform advancement; (**c**) Complete filling.

**Figure 22:**
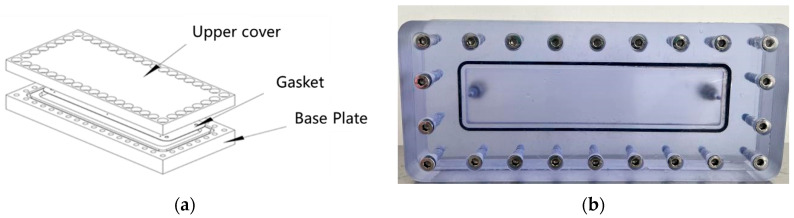
Visualized vertical fracture model: (**a**) Schematic; (**b**) Physical model.

Conventional polymer gel plugging systems are susceptible to gravitational settling during migration within fractures, advancing at an inclination angle that leads to incomplete filling and eventual plugging failure. In contrast, when the thixotropic gel migrates through a single vertical fracture, gravitational settling is minimal. The gel front advances uniformly, achieving complete fracture filling, as illustrated in Figure 21. A visualized vertical fracture model (Figure 22) was employed to investigate the self-filling behavior of the thixotropic polymer gel system in formation fractures. The fracture model was filled with water to simulate formation fluid properties. The model was oriented vertically (90° inclination) with a 5 mm fracture width, and gel was injected at a constant rate of 10.0 mL/min via a horizontal flow pump.

**Figure 23:**
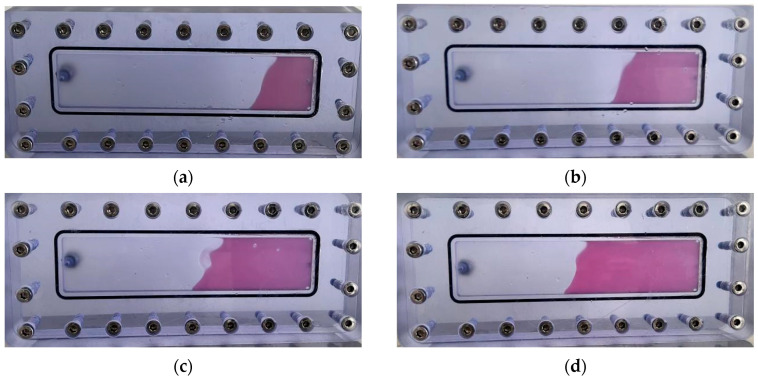

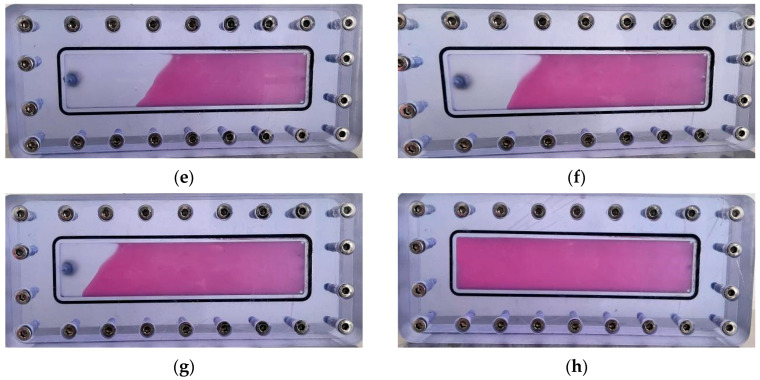
Self-filling progression at different injection volumes: (**a**) 0.2 PV; (**b**) 0.3 PV; (**c**) 0.4 PV; (**d**) 0.5 PV; (**e**) 0.6 PV; (**f**) 0.7 PV; (**g**) 0.8 PV; (**h**) 1.0 PV.

As illustrated in Figure 23, the thixotropic polymer gel system exhibits progressive self-filling within the 5 mm-wide fracture. At 0.2 PV injection volume, gel primarily concentrates near the fracture front with limited spatial distribution. When injection reaches 0.4 PV, the trapezoidal migration front remains confined to the forward section, displaying tortuous morphology without extending to the fracture top. Increasing injection to 0.6 PV drives gel advancement toward the mid-fracture region at a 45° angle relative to the bottom plate, covering approximately half the fracture space. At 0.8 PV, significant expansion occurs with the front, middle, and rear sections becoming predominantly filled, though minor unfilled zones persist near the top. Complete uniform filling throughout the vertical fracture is achieved with a 1.0 PV injection volume.

Quantitative filling analysis (Table 3) confirms minimal gravitational settling effects in vertical fractures, with filling efficiency progressively increasing from 24% at 0.2 PV to 100% at 1.0 PV. The gel front advances uniformly within the fracture model, demonstrating negligible gravity sensitivity and complete dimensional adaptability. This behavior stems from the synergistic action of the post-gelation 3D network structure and rigid inorganic particles from the resin hardener, which collectively enhance matrix rigidity and regulate flow dynamics. Operationally, the system leverages its intrinsic shear-thinning rheology: under high shear during injection, apparent viscosity drops significantly to facilitate pumpability into fractures; upon entering the fracture space, reduced shear restores high viscosity, enabling uniform migration and complete plugging of loss channels without preferential flow paths.

#### 2.2.6. Plugging Performance of Polymer Gel System

The pressure-bearing plugging capacity of the optimized thixotropic gel was evaluated using a high-temperature/high-pressure loss circulation displacement device. After injecting the gel solution into sand-packed tube models, samples were aged at 120 °C and 140 °C for 1 d and 3 d. Post-gelation, water or nitrogen sources were connected to the inlet, while pressure sensors monitored breakthrough pressure in real-time. Eight barite-weighted samples (density: 1.5 g·cm^−3^) underwent comparative testing under identical aging conditions. Breakthrough pressure, the critical metric for plugging performance, indicates structural failure when the gel barrier is compromised. Higher breakthrough pressures sustained over extended durations signify superior plugging capability.

As shown in Table 4, after 1 day aging at 120 °C, the gel exhibited plugging capacities of 166 kPa/m (water flooding) and 122 kPa/m (gas flooding). After 3 days, capacities decreased to 148 kPa/m (water) and 100 kPa/m (gas). At 140 °C, pressure-bearing capacity slightly declined. Notably, breakthrough pressures for nitrogen were consistently lower than for water due to gas molecules penetrating gel micropores more readily. This confirms superior liquid-plugging efficacy.

**Figure 24:**
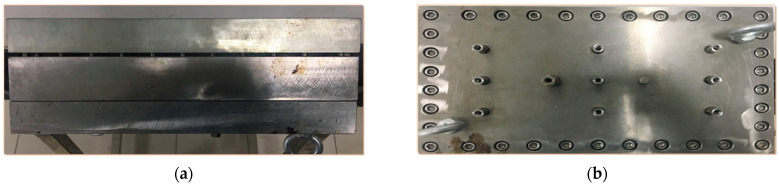
Schematic of fracture plate model: (**a**) Side view; (**b**) Top view.

Fracture plugging performance was further assessed using a high-pressure fracture plate model (20 cm length × 10 cm height, 5–10 mm adjustable width; Figure 24). After injecting the gel into vertical fractures and aging at 120 °C, water flooding tests measured breakthrough pressures. The gel effectively plugged fracture inlets/outlets, forming a highly intact, viscous barrier under low shear. Minimal gravitational settling enabled uniform distribution, creating an elastic plug. Results (Table 5) demonstrate an inverse correlation between fracture width and breakthrough pressure: narrower outlets (6 mm) achieved 105.6 kPa capacity, while wider fractures (10 mm) registered 69.7 kPa, confirming effective plugging across variable fracture dimensions.

## 3. Conclusions

(1) This study introduced the concept of thixotropy to develop a novel polymer gel plugging system. Through optimization of polymer, crosslinker, and resin hardener types/concentrations, the gel formulation was refined to the following: 1% polymer J-1 + 0.3% catechol + 0.6% hexamethylenetetramine (HMTA) + 15% resin hardener.
(2) Rheological characterization demonstrated exceptional performance: At 120 °C, apparent viscosity reached 1471 mPa·s under low shear (0.1 s^−1^) and maintained 931 mPa·s at high shear (100 s^−1^), confirming superior shear resistance. Hysteresis in cyclic shear tests validated excellent thixotropy through reversible structure breakdown/rebuilding. After 9 days of aging at high temperatures, minimal strength reduction (G′ decreased from 17.5 to 16 Pa) demonstrated outstanding thermal stability.
(3) The engineered system exhibited uniform migration with negligible gravitational effects, enabling complete filling of fractures across dimensions. Plugging capacities of 166 kPa/m (water flooding) and 122 kPa/m (gas flooding) after 1 day of aging at 120 °C, with significantly enhanced pressure-bearing capability when weighted. Effective plugging in vertical fractures, achieving 105.6 kPa pressure capacity in 6 mm fractures through the formation of intact gel barriers. This thixotropic polymer gel system provides a technically robust solution for controlling lost circulation in challenging high-temperature formations.

## 4. Experimental Materials and Methods

### 4.1. Experimental Materials

#### 4.1.1. Experimental Reagents

The polymer materials used were J-1, J-2, J-3, and J-4 from Shandong Nor Chemical Co., Ltd., Dongying, China; the molecular weights of ZP-1 and ZP-2 are 700–900 and 800–1000 Daltons, respectively. The aldehyde-based crosslinking agents used were formaldehyde and paraformaldehyde from Shanghai Aladdin Reagent Co., Ltd., Shanghai, China., and hexamethylenetetramine (HMTA) from Shanghai Macklin Biochemical Technology Co., Ltd., Shanghai, China. The phenolic crosslinking agents used were resorcinol, hydroquinone, and catechol from Shanghai Macklin Biochemical Technology Co., Ltd., Shanghai, China. Formaldehyde, paraformaldehyde, HMTA, resorcinol, hydroquinone, and catechol are pure AR. The resin curing agent used was urea–formaldehyde resin from Zibo Ocean Industrial Co., Ltd., Zibo, China. Deionized water prepared in the laboratory was used as the solvent.

#### 4.1.2. Experimental Instruments

The experimental main instruments are listed in Table 6:

### 4.2. Experimental Methods

#### 4.2.1. Preparation of Polymer Solution

The polymer stock solution was prepared by accurately weighing vacuum-dried polymer powder and dissolving it in deionized water to achieve a 1.0% concentration. Using a magnetic stirrer (300 ± 50 rpm at 25 ± 0.5 °C), the mixture was stirred for ≥6 h to ensure complete dissolution, followed by 24 h of static aging at 25 °C. Serial dilutions to target concentrations were performed with the same batch of deionized water, with thorough mixing at each dilution stage.

First, slowly pour the 0.3% catechol solution into the 1% polymer J-1 mother liquor under low-speed stirring at 300 rpm. After the mixture turns into a light brown transparent state, add 0.6% hexamethylenetetramine (HMTA) while reducing the stirring speed to 200 rpm until the solution exhibits a pale-yellow opalescent appearance. Finally, incorporate a 15% resin curing agent into the main system and mix thoroughly to form the polymeric gel system.

All experimental data represent precise results averaged from triplicate experiments.

#### 4.2.2. Evaluation of Gelling Time and Strength

The polymer gel solution was transferred into temperature/pressure-resistant bottles and placed in a constant-temperature drying oven for crosslinking reactions under high temperature. Gelling time and strength were assessed using the Sydansk bottle test method [32]. Gelling time was recorded when gel state stabilized, and gel strength was classified into nine grades [33] based on inverted suspension and protrusion states: A (discontinuous), B (highly fluid), C (fluid), D (moderately fluid), E (slightly fluid), F (highly deformable non-fluid), G (moderately deformable non-fluid), H (slightly deformable non-fluid), I (rigid). All experimental data represent precise results averaged from triplicate experiments.

#### 4.2.3. Rheological Strength Testing

Rheological characterization of the polymer gel system was conducted using a plate rheometry platform based on the HAKKE Mars60 rheometer (Thermo Fisher Scientific, Germany). The experimental configuration employed a P35 titanium alloy coaxial rotor with a controlled plate gap of 52 μm. Dynamic rheological analysis was performed by combining steady-state shear scanning (shear rate range: 0.1–100 s^−1^) and dynamic oscillatory frequency scanning (fixed frequency: 1 Hz; stress range: 0.1–100 Pa). This dual-mode approach systematically investigated the structural strength and viscoelastic response characteristics of the gel network. All experimental data represent precise results averaged from triplicate experiments.

#### 4.2.4. Microstructural Analysis

Microstructural morphology was characterized using a JSM-7200F field-emission environmental SEM (FEI, USA). Gel samples were cryo-fixed in liquid nitrogen, lyophilized to sublime water, mounted on conductive tape, and gold-sputtered. Images were acquired at room temperature with representative 10 μm scale bars. All experimental data represent precise results averaged from triplicate experiments.

#### 4.2.5. FTIR Characterization

Fourier Transform Infrared Spectroscopy (Shanghai Precision Instrument Co., Ltd. Shanghai, China) (400–4000 cm^−1^, 35 scans) analyzed molecular structures. Dried gel samples (1.0 wt%) were mixed with KBr powder, ground, and pressed into optical-grade pellets. Functional groups and chemical bonds were identified via characteristic peaks (e.g., C=O stretch, N–H bend). All experimental data represent precise results averaged from triplicate experiments.

#### 4.2.6. Thermogravimetric Analysis (TGA)

Thermal stability was evaluated using a TGA 2 SF analyzer (METTLER TOLEDO, Switzerland). Samples were heated from 30 to 600 °C at 10 °C/min under 300 Pa pressure with 20 cm^3^/min N_2_ flow. All experimental data represent precise results averaged from triplicate experiments.

#### 4.2.7. Nuclear Magnetic Resonance (NMR)

^13^C-NMR spectroscopy resolved molecular configurations. Samples were dissolved, loaded into NMR tubes, and analyzed after shimming for magnetic field homogeneity. Pulse sequence parameters were optimized for carbon spectrum acquisition. All experimental data represent precise results averaged from triplicate experiments.

#### 4.2.8. Plugging Performance Evaluation

The plugging effectiveness of the gel lost circulation material was evaluated using a high-temperature/high-pressure (HTHP) lost circulation simulator. A visualized vertical fracture model (3 mm width) was employed to examine the self-filling behavior of the thixotropic polymer gel system within formation fractures. Simultaneously, plugging performance was quantitatively assessed using a sand-packed tube model (50 cm length × 38 mm inner diameter) with 6 mm inlets/outlets. Water was injected at a constant rate of 5 mL/min, while pressure sensors recorded real-time pressure differentials and maximum breakthrough pressure between the model’s inlet and outlet. Since breakthrough pressure directly correlates with gel strength (higher gel strength yields greater breakthrough resistance), measuring breakthrough pressures at various time intervals enabled a systematic investigation of the high-temperature/high-pressure plugging efficacy of the thixotropic polymer gel system. All experimental data represent precise results averaged from triplicate experiments.

## Figures and Tables

**Table 1:** Effect of different aldehyde crosslinkers on gel system performance.

Aldehyde Crosslinker and Concentration (%)		Catechol Concentration (%)	Gelling Time (h)	Gelation Quality and Stability
Formaldehyde	0.1	0.3	2	Gel strength reached Grade G; Severe dehydration and degradation after 6 h aging
	0.3		1	Gel strength reached Grade G; Severe dehydration and degradation after 5 h aging
	0.6		1	Gel strength reached Grade G; Severe dehydration and degradation after 5 h aging
Paraformaldehyde	0.1	0.3	No gelation	-
	0.3		No gelation	-
	0.6		No gelation	-
HMTA	0.1	0.3	15	Gel strength Grade G; <10% dehydration after 7 d
	0.3		11	Gel strength Grade H; <10% dehydration after 7 d
	0.6		6	Gel strength Grade H; <10% dehydration after 7 d

**Table 2:** Effect of different phenolic crosslinkers on gel system performance.

Crosslinker and Concentration (%)	Polymer Concentration (%)	Gelling Time (h)	Gelation Quality and Stability
0.3% Phenol + 0.6% HMTA	0.2	-	Strength too weak
	0.4	-	Strength too weak
	0.6	-	Strength too weak
	0.8	-	Strength too weak
	1.0	-	Strength too weak
0.3% Hydroquinone + 0.6% HMTA	0.2	15	Gel strength Grade D; <35% dehydration after 7 d
	0.4	14	Gel strength Grade D; <35% dehydration after 7 d
	0.6	12	Gel strength Grade D; <35% dehydration after 7 d
	0.8	10	Gel strength Grade E; <30% dehydration after 7 d
	1.0	8	Gel strength Grade E; <30% dehydration after 7 d
0.3% Catechol + 0.6% HMTA	0.2	16	Gel strength Grade D; <10% dehydration after 7 d
	0.4	14	Gel strength Grade D; <10% dehydration after 7 d
	0.6	11	Gel strength Grade E; <10% dehydration after 7 d
	0.8	8	Gel strength Grade F; <10% dehydration after 7 d
	1.0	6	Gel strength Grade H; <10% dehydration after 7 d

**Table 3:** Filling efficiency of thixotropic gel in vertical fractures.

Fracture Width	Injection Volume	Primary Filling Zone	Filling Degree
5 mm	0.2 PV	Mid-fracture	24%
	0.3 PV	Mid-fracture	32%
	0.4 PV	Mid-fracture	45%
	0.5 PV	Mid-fracture	53%
	0.6 PV	Bottom	62%
	0.7 PV	Bottom	75%
	0.8 PV	Bottom	90%
	1.0 PV	Mid-fracture	100%

**Table 4:** Plugging the strength of the gel system.

Sample	Length (cm)	Temp (°C)	Aging (d)	Pressure Gradient. (kPa/m)
1 (Water flood)	50	120	1.0	166
2 (Water flood)	50	120	3.0	148
3 (Water flood)	50	140	1.0	142
4 (Water flood)	50	140	3.0	108
5 (Gas flood)	50	120	1.0	122
6 (Gas flood)	50	120	3.0	100
7 (Gas flood)	50	140	1.0	84
8 (Gas flood)	50	140	3.0	66

**Table 5:** Plugging strength in fracture plate models.

ID	Fracture Width (mm)	Plugging Strength (kPa)
1	6	105.6
2	8	88.3
3	10	69.7

**Table 6:** Main Instruments.

ID	Instrument Name	Manufacturer
1	TGA/DTA Thermogravimetric Analyzer	METTLER TOLEDO (Greifensee, Switzerland)
2	High-Temperature/High-Pressure Leak-Plugging and Displacement Apparatus	Nantong Xinhua Cheng Scientific Instrument Co., Ltd. (Nantong, China)
3	Quanta 200F Field Emission Scanning Electron Microscope (FE-SEM)	FEI (Morristown, NJ, USA)
4	HAAKE MARS 60 Rheometer	Thermo Fisher Scientific (Dreieich, Germany)
5	Fourier Transform Infrared Spectrometer (FTIR-7600)	Shanghai Precision Instrument Co., Ltd. (Shanghai, China)
6	Constant Temperature Blast Drying Oven	Beijing Heng Aode Instrument (Beijing, China)
7	Visualized Fracture Model	Nantong Xinhua Cheng Scientific Instrument Co., Ltd. (Nantong, China)
8	Magnetic Stirrer	Shanghai NIYUE Instrument Co., Ltd. (Shanghai, China)

## Data Availability

Data are contained within the article.
